# The Chromosomal Constitution of Embryos Arising from Monopronuclear Oocytes in Programmes of Assisted Reproduction

**DOI:** 10.1155/2014/418198

**Published:** 2014-05-06

**Authors:** Bernd Rosenbusch

**Affiliations:** Department of Gynaecology and Obstetrics, University of Ulm, Prittwitzstraße 43, 89075 Ulm, Germany

## Abstract

The assessment of oocytes showing only one pronucleus during assisted reproduction is associated with uncertainty. A compilation of data on the genetic constitution of different developmental stages shows that affected oocytes are able to develop into haploid, diploid, and mosaic embryos with more or less complex chromosomal compositions. In the majority of cases (~80%), haploidy appears to be caused by gynogenesis, whereas parthenogenesis or androgenesis is less common. Most of the diploid embryos result from a fertilization event involving asynchronous formation of the two pronuclei or pronuclear fusion at a very early stage. Uniparental diploidy may sometimes occur if one pronucleus fails to develop and the other pronucleus already contains a diploid genome or alternatively a haploid genome undergoes endoreduplication. In general, the chance of obtaining a biparental diploid embryo appears higher after conventional in vitro fertilization than after intracytoplasmic sperm injection. If a transfer of embryos obtained from monopronuclear oocytes is envisaged, it should be tried to culture them up to the blastocyst since most haploid embryos are not able to reach this stage. Comprehensive counselling of patients on potential risks is advisable before transfer and a preimplantation genetic diagnosis could be offered if available.

## 1. Introduction

The technology of assisted reproduction aims at achieving oocyte fertilization by incubation of cumulus-intact oocytes in the presence of a defined number of motile spermatozoa (conventional in vitro fertilization, IVF) or by injection of single spermatozoa into denuded, cumulus-free oocytes (intracytoplasmic sperm injection, ICSI). Both procedures are followed about 16 to 20 hours later by the so-called pronucleus check. Here, successful and normal fertilization is identified by the appearance of two pronuclei (PN) in the ooplasm and detection of two polar bodies in the perivitelline space, whereas the presence of more than two PN is considered to be associated with genetic disorders, mostly triploidy [1]. Consequently, these multipronuclear oocytes are excluded from further cell culture and embryo transfer. In contrast, recommendations on the treatment of oocytes displaying only one pronucleus are accompanied by greater uncertainty. In case of parthenogenetic activation, one should expect the formation of a haploid embryo with exclusively maternal chromosomes and therefore transfer should be cancelled. If, however, the PN had appeared asynchronously or underwent an undetected fusion, diploid biparental and transferable embryos may be available. In fact, a few pregnancies have been reported after transfer of embryos developing from monopronuclear oocytes [2–6].

The frequency of monopronuclear oocytes among all pronuclear stages has reached 7.7% after IVF and 5.0% after ICSI in a large study evaluating more than 6,000 cells for each technique [7]. Information on the chromosomal constitution of the resulting embryos appears to be of clinical interest particularly in rare cases without regular formation of two PN. The present report therefore summarizes pertinent data, reviews possible mechanisms of origin of a single pronucleus, and tries to deduce recommendations for handling affected oocytes during assisted reproduction.

## 2. Material and Methods

The literature search for this review is based on PubMed and Scopus and includes results found until the end of January 2014. The key words used were “single pronucleus,” “monopronuclear,” “monopronucleus,” “single pronucleated,” “unipronucleate,” “unipronuclear,” “one-pronuclear,” and “single-nucleated”, each in combination with “oocyte(s),” “zygote(s),” and “embryo(s).” Each identified article was checked for the relevant secondary literature. If specific data were excluded from the compilation of results, the reasons have been explained in the corresponding section.

**Figure 1:**
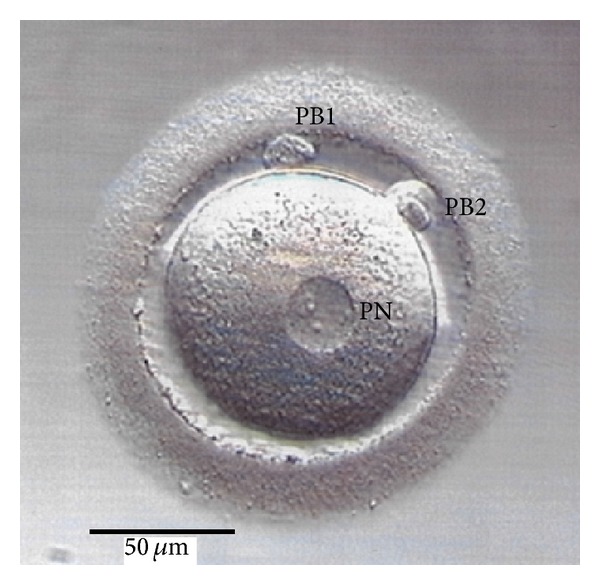
Following ICSI in our programme of assisted reproduction, this oocyte displayed a single pronucleus (PN) and two polar bodies (PB1 and PB2) on the following morning. Because another pronucleus could not be detected after a second inspection several hours later, the cell was subjected to cytogenetic analysis and revealed a haploid karyotype (23,X).

The cited studies have examined different developmental stages, including monopronuclear oocytes, zygotes, and embryos up to the blastocyst. It should be noted that a monopronuclear female gamete (Figure 1) will undergo breakdown of the pronuclear membrane after DNA replication and hence the pronucleus will disappear comparable to the situation in normally fertilized bipronuclear oocytes. The next stage is the zygote though, strictly speaking, this description does not apply to parthenogenetically activated cells because a zygote is defined to result from the union of two haploid gametes and should therefore always contain a diploid chromosome set. However, the common nomenclature has been maintained in the present review because some zygotes indeed turned out biparental diploid (see Section 3).

For cytogenetic analysis, the above-mentioned developmental stages were frequently incubated in the presence of chemicals that block mitosis, for example, colcemid. The cells were then fixed on glass slides and the chromosomes were stained in order to establish karyotypes or allow at least chromosome counting. Some zygotes that developed from monopronuclear oocytes have been fixed during our cytogenetic investigations of unfertilized and abnormally fertilized female gametes. This project had been approved by the ethical committee of the University of Ulm and details of our technique have been described elsewhere [8]. Briefly, we used a mixture of podophyllotoxin and vinblastine instead of colcemid, a gradual fixation air-drying method and homogeneous Giemsa staining of the chromosomes. The corresponding cytogenetic results included in the present review have not been published before.

Fluorescence in situ hybridization (FISH) is another approach to examine cells that had been fixed on a glass slide. The method can be applied to interphase nuclei and therefore preceding exposure to colcemid is not necessary. The most frequently used DNA probes are those for chromosomes X, Y, 18, and 13/21. FISH has been applied to intact developmental stages but also to single biopsied cells from embryos [3, 9, 10]. Levron et al. [11] isolated the karyoplast, that is, the nucleus with a small amount of cytoplasm from the remaining cytoplast in monopronuclear oocytes to analyze them separately. In one instance, polymerase chain reaction (PCR) was used in combination with FISH [9].

van der Heijden et al. [12] presented a technique based on the asymmetrical distribution of histone modifications in male and female PN. Histones are DNA-associated proteins and determination of the presence of methylated lysine residues at a certain position of the N-terminal tail of histone H3 by a specific antibody allows distinguishing paternal and maternal chromatin because only the latter will be stained. The method yielded information on the haploid or diploid state of monopronuclear zygotes and the parental origin of the PN but aneuploidy could not be assessed.

## 3. Results

Relevant information on the genetic constitution of monopronuclear oocytes and resulting developmental stages has been outlined in Table 1 according to their origin (conventional IVF or ICSI). The data provided by Balakier et al. [13] for cleavage stages have been excluded because of the low number of analyzable cells in each category (four embryos, one morula, and two blastocysts). The investigation of Munné et al. [9] was not considered because the authors themselves admitted that the applied technique would not allow clear distinction of monosomy and haploidy or trisomy and triploidy. Moreover, these authors [9] stated that “an X result by PCR could be either a haploid cell, a female diploid cell, or a trisomic or triploid female cell.” From the study of Lim et al. [14], only the conventionally karyotyped embryos were included. The number of cells analyzed by FISH (*n* = 14) was low and subdividing them further (IVF or ICSI, zygotes or embryos) would have yielded very small groups without providing additional information. The study of Campos et al. [10] was excluded because diploid-aneuploid and haploid-aneuploid cases could not be distinguished.

Further difficulties encountered when trying to classify the results particularly concern cleavage stages with a larger number of analyzable blastomeres. These often show a coexistence of diploidy, haploidy, polyploidy, and superimposed numerical chromosome abnormalities. Presenting details would have been too confusing and therefore the cytogenetic terms chosen for Table 1 (“haploid,” “diploid,” and “other”) are simplifications. In other words, the categories “diploid” and “haploid” also contain cells with deviations from the respective exact chromosome count of 46 or 23. For instance, the diploid-aneuploid embryos and blastocysts listed by Liao et al. [15] have been counted as diploid. Concerning the study of Mateo et al. [16], it was decided to classify diploid-mosaic cells as diploid, haploid-mosaic cells as haploid, and the remainder as carrying “other” aberrations. Since the main intention of the present review was to differentiate fertilized from unfertilized cells, this subjective approach appeared justifiable. The possible biparental origin of diploid cells was not considered in Table 1 but will be addressed below.

### 3.1. Oocytes and Uncleaved Zygotes

The genetic constitution of monopronuclear oocytes and zygotes obtained after IVF has been examined in three studies [11–13] and it was shown that 37.5% to 86.7% of the cells were diploid (Table 1). This incidence is conspicuously different from the range of 0% to 30.3% detected in monopronuclear cells produced by ICSI ([12, 17], own unpublished results). Particularly the study of Macas et al. [17] and our own unpublished investigation, both applying conventional cytogenetics to uncleaved zygotes, failed to reveal a case of diploidy. In our material, 4 out of 18 haploid cells (22.2%) carried a chromosomal aberration (hypo- or hyperhaploidy, see Table 2), whereas Macas et al. [17] reported an incidence of 32.1%. Here, it should be noted that cytogenetic investigations of monopronuclear zygotes are surprisingly scarce in view of the fact that these cells can inform about the total incidence of aneuploidy arising from female meioses I and II. Finally, 15 out of the 18 zygotes (83.3%) examined by us showed prematurely condensed sperm chromosomes comparable to the patterns found in unfertilized oocytes [18], indicative of correct sperm insertion into the ooplasm during ICSI.

### 3.2. Embryos and Blastocysts

As shown in Table 1, eight studies investigated the chromosomal constitution of embryos or blastocysts after IVF [2, 3, 7, 14, 19–22]. ICSI-derived embryos or blastocysts were included in five reports [3, 7, 14, 16, 21]. According to these data, the incidence of diploid IVF embryos varies between 13.0% [19] and 80.5% [2] and is again considerably lower after ICSI with rates between 2.2% [16] and 37.5% [14]. Whereas all six IVF blastocysts examined by Otsu et al. [22] were diploid or diploid mosaics, only three of eight (37.5%) ICSI-derived blastocysts analyzed by Mateo et al. [16] revealed diploidy. However, it is evident that both observations are based on a low number of cases. The results of Liao et al. [15] had to be considered separately because the origin of the embryos (IVF or ICSI) was not specified. Nevertheless, an important point in their study is that the rate of diploidy increases from 38.9% in early cleavage stages to 78.0% in blastocysts.

### 3.3. A Closer Look at Diploid Stages

As soon as a monopronuclear oocyte undergoes further development into a diploid zygote or even an embryo, it should be ascertained whether the diploid condition was actually caused by fertilization (= biparental or heteroparental diploidy) or by specific mechanisms giving rise to uniparental diploidy, for instance, endoreduplication of the haploid female chromosome set. Pertinent information on this topic has been summarized in Table 3. Investigating histone modifications in monopronuclear zygotes, van der Heijden et al. [12] regarded the presence of two chromatin domains with a nonuniform staining pattern as proof of a biparental origin. According to this approach, all 39 diploid IVF zygotes and all 10 diploid ICSI zygotes were classified as biparental because male and female chromatin were detected. More common, however, is to determine the presence of a Y-chromosome as evidence for sperm penetration. After conventional IVF, a minimum of 40% of diploid embryos had a Y-chromosome [20] but this incidence even reached 66.7% both in isolated karyoplasts [11] and in blastocysts [22]. After ICSI, the frequency of diploid embryos with a Y-chromosome ranged from 16.7% [3] to 52.2% [21]. Mateo et al. [16] found a Y-chromosome in 19/54 (35.2%) ICSI embryos but these data could not be included in Table 3 because it was not clear whether mosaic haploid embryos were involved. Finally, it should be added that 15/31 (48.4%) complex mosaic IVF embryos and 12/16 (75.0%) complex mosaic ICSI embryos revealed a Y-chromosome [7]. The latter authors [7] also reported one diploid ICSI embryo with a YY-chromosome constitution. Taken together, these figures support the former statement by Munné et al. [9] who, having found a Y-chromosome in 41% (9/22) of the embryos, suggested that approximately 80% may have originated from fertilized eggs. The authors [9] arrived at this value by doubling the percentage of Y-bearing embryos because it is assumed that X- and Y-spermatozoa participate equally in fertilization. With this formula in mind, some of the data shown in Table 3 suggest an even higher incidence of biparental diploidy that may reach nearly 100% independent of IVF or ICSI, whereas uniparental diploidy in cleavage stages arising from monopronuclear oocytes appears to be an exception.

## 4. Discussion

Up to now, the transfer of embryos that developed from monopronuclear IVF oocytes resulted in one pregnancy with unknown outcome [3], the birth of two healthy children and one biochemical pregnancy [2], and the birth of a normal healthy boy [5]. Moreover, even the birth of normal twin boys following transfer of a single embryo has been reported [6]. In contrast, only Barak et al. [4] achieved the birth of a normal healthy boy after round spermatid injection accompanied by formation of one pronucleus. It should also be mentioned that a diploid (46,XX) human embryonic stem cell (hESC) line could be derived from a monopronuclear ICSI zygote [23], whereas another group [15] established 33 hESC lines. The latter authors [15] who did not indicate the origin of the monopronuclear oocytes (IVF or ICSI) obtained a diploidy rate of 97% (32/33) and only one abnormal (47,XY,+16) cell line. In contrast to these successes, Petignat et al. [24] described a twin pregnancy combining a complete hydatidiform mole and normal pregnancy that had to be terminated. This pregnancy occurred after transfer of two embryos, one obtained from a normally fertilized oocyte with two PN and the other from a monopronuclear oocyte. The authors [24] hypothesized that the oocyte with one pronucleus gave rise to the hydatidiform mole and emphasized the danger of transferring the corresponding embryos. The underlying mechanism in this peculiar case would involve fertilization by a haploid spermatozoon with subsequent chromosome duplication or fertilization by a diploid spermatozoon, always accompanied by failed formation of the female pronucleus. This annotation shows that it must be clarified which mechanisms are responsible for the different genetic compositions, particularly haploidy and diploidy.

### 4.1. Haploid Embryos

Haploidy is generally attributed to parthenogenesis, gynogenesis, or androgenesis and these terms have been explained in detail elsewhere [25, 26]. Briefly, parthenogenesis means the development of an embryo from an oocyte without any intervention of a male gamete. Oocytes can, for instance, be activated by heat or mechanical means. It is evident that these embryos contain only the female genome. The same is true in the case of gynogenesis but here the oocyte has been stimulated by a spermatozoon to undergo the second meiotic division. Cleavage then proceeds without participation of the male genome. On the other hand, androgenesis also starts with oocyte activation by a spermatozoon but the female genome will be genetically inactivated or completely extruded and only the male genome is involved during subsequent development.

About 45% of monopronuclear IVF zygotes showed signs of sperm penetration [13] and the authors surmised that, in oocytes without visible sperm heads or nucleus-like structures, the sperm chromatin might have undergone complete disintegration or extrusion to form observed but undefined “extra bodies.” Thus, the incidence of sperm penetration could even be higher. More data are available for monopronuclear ICSI zygotes. Here, intact sperm heads or decondensed sperm chromatin was found in 76 to 86.5% of examined cases [27–29]. Our own unpublished results of 83.3% are in good agreement with these figures. In one study [29], a male origin of the single pronucleus was determined in only 4% of the examined oocytes due to the presence of a sperm tail and it was assumed that the entire maternal chromatin had been extruded into a polar body (PB) or did not succeed in forming a pronucleus. Oocytes in which the meiotic spindle cannot be detected at the time of ICSI appear to be more susceptible to formation of a single male pronucleus [30]. Assessing histone methylation patterns in uncleaved zygotes, van der Heijden et al. [12] arrive at different figures. In their study, only paternal chromatin was found in 24.2% of ICSI zygotes and in 4.4% of IVF zygotes. These authors concluded that complete extrusion of the maternal chromatin during formation of the second PB might be a quite frequent event. It remains to be determined whether the applied detection methods are responsible for the varying rates of male PN reported by Kovacic and Vlaisavljevic [29] and van der Heijden et al. [12] for ICSI zygotes.

In the discussion on causative mechanisms, ICSI could indeed be regarded as a separate phenomenon because it involves both a mechanical stimulus and participation of a spermatozoon that is inserted into the ooplasm. Most probably, however, a sperm factor activates the oocyte and then sperm chromatin decondensation stops [29], whereas female pronucleus formation proceeds normally. This would again comply with the definition of gynogenesis. Taken together, most of the available data support the opinion that the majority (~80%) of haploid monopronuclear oocytes and resulting embryos are produced by gynogenesis and that parthenogenesis or androgenesis is less common.

### 4.2. Biparental Diploid Embryos

An asynchronous appearance of PN is the first possibility to explain the existence of biparental diploidy in embryos that develop from monopronuclear oocytes. For instance, Staessen et al. [2] performed a second observation of 312 single-pronucleated oocytes 4 to 6 hours after the initial assessment and detected a second pronucleus in 25% of these cases. The authors concluded that a single observation of an oocyte with one pronucleus does not allow differentiating between asynchrony of pronuclear development and parthenogenetic activation. Since delayed formation of the second pronucleus may not be a rare event, monopronuclear oocytes should therefore be rechecked after some hours. Consequently, a proportion of diploid embryos could have arisen from fertilized oocytes in which the asynchronous pronuclear formation had been overlooked. These embryos would be characterized by a diploid, 46,XX or 46,XY chromosome constitution.

However, how does diploidy arise if definitely only one pronucleus persists during the whole observation period? A solution is offered by the concept of pronuclear fusion that has been put forward by Levron et al. [11]. These authors suggested that monospermic diploid monopronuclear zygotes may be formed by a fusion of the paternal and maternal genomes during syngamy, most probably by very early enclosure in a common pronuclear envelope rather than by fusion of pronuclear membranes at a later stage. It was further assumed that sperm penetration close to the metaphase plate of the oocyte might predispose to this modified fertilization process. Pronuclear fusion was not observed in an investigation using time-lapse video cinematography [31] but the number of examined oocytes (43 with formation of PN) appears too low for definite conclusions. Whereas Levron et al. [11] did not address the question whether fused PN possibly show an increase in size, Otsu et al. [22] differentiated between large (29–34 *μ*m) and small (23–26 *μ*m) PN. Only some oocytes (6/34) from the group with larger PN were able to reach the blastocyst stage and these blastocysts were diploid or diploid mosaic. Otsu et al. [22] suggested that larger PN might be a product of pronuclear fusion before nuclear membrane breakdown. In contrast, others [16] could not demonstrate a correlation between pronuclear size and chromosomal constitution and also denied the existence of pronuclear fusion at a later stage. The only possibility for formation of a diploid heteroparental pronucleus would consist in an irregular membrane formation enclosing maternal and paternal genomes [16].

Prior to these investigations, however, Tesarik and Mendoza [32] had reported that oocytes injected with spermatids may develop two PN that later fuse to form a “syngamy nucleus.” This nucleus was described to be only slightly larger than a pronucleus. It is currently not clear whether the described phenomenon is restricted to the use of sperm precursor cells for injection. Further confirmatory observations are rare and spermatid injections have apparently been abandoned during the past years. Obviously, there is a need for more basic research concerning the dynamics of pronuclear development, fusion events, and pronuclear size. Comparable to cases of asynchronous pronuclear formation, embryos resulting from fertilization and early fusion of the genomes or later pronuclear fusion should reveal a diploid, 46,XX or 46,XY chromosome constitution.

### 4.3. Uniparental Diploid Embryos

An embryo within this category can carry a diploid 46,XX genome which is exclusively composed of female chromosomes as soon as a diploid oocyte starts to cleave without participation of a male genome. If on the other hand the genome is exclusively derived from the male gamete, the following chromosome complements can occur in embryos: 46,XY (first meiotic nondisjunction during spermatogenesis) and 46,XX or 46,YY (second meiotic nondisjunction). In each case, a diploid spermatozoon would fertilize an oocyte and cleavage would commence without participation of the female genome. As already mentioned above, Staessen and van Steirteghem [7] detected one diploid ICSI embryo with two Y-chromosomes. This observation can be explained by a failed formation of the female pronucleus and development of a diploid male pronucleus due to injection of a diploid spermatozoon. Alternatively, however, injection of a haploid spermatozoon might have been accompanied by suppression of the female and endoreduplication in the male pronucleus. Endoreduplication has been reported to affect not only single chromosomes but also complete chromosome sets and may in the latter case contribute to the development of triploidy if it occurs in one of the two PN of a regularly fertilized oocyte [33]. It is therefore conceivable that endoreduplication in a monopronuclear oocyte produces uniparental diploid embryos but clear evidence for this assumption is lacking.

Nonextrusion of the second PB has been discussed as another mechanism that may cause uniparental diploidy in embryos arising from monopronuclear oocytes [19]. In such cases, the 23 oocyte chromosomes should separate into single chromatids but all 46 chromatids will remain within the ooplasm, become enclosed by a pronuclear membrane, and undergo DNA replication, thus restoring a diploid female chromosome set. A male pronucleus will not be formed. This concept has been described as one of the mechanisms for diploid parthenogenesis [26] but it appears questionable in view of the findings for tripronuclear ICSI oocytes. Here, it is generally accepted that nonextrusion of the second PB leads to two individual haploid female PN and not to a single diploid female pronucleus [33]. More data are therefore needed to verify a participation of the second PB in producing monopronuclear diploid oocytes and embryos.

From these considerations, it becomes evident that a variety of mechanisms can influence the genetic composition of zygotes and embryos obtained from monopronuclear oocytes. In addition, mitotic nondisjunction of single chromosomes or whole chromosome sets may occur in cleavage stages and thus explain the observation of polyploid, mosaic, complex, and chaotic cases.

### 4.4. Additional Remarks

From the preceding compilation of published results, two important points can be condensed: (a) monopronuclear oocytes are able to develop into embryos with variable chromosomal constitutions and (b) the majority of diploid embryos obviously result from a fertilization event. How should clinicians proceed with monopronuclear oocytes in view of this conflicting information? First, monopronuclear oocytes should be rechecked after the first assessment of pronuclear formation to detect delayed appearance of a second pronucleus. If this is not the case, one may follow Sultan et al. [3] who recommended that embryos developing from monopronuclear IVF oocytes may be replaced, whereas those obtained after ICSI would not be suitable. Nowadays, however, the frequently used transfer of blastocysts may provide an additional option. As discussed by Feenan and Herbert [1], human parthenotes are capable of cleaving to the 8-cell stage but they rarely seem to develop up to the blastocyst (Table 1). Liao et al. [15] added that, besides haploidy, autosomal aneuploidy and polyploidy were eliminated in blastocysts and they concluded that blastocyst formation would be a useful indicator for normal fertilization and chromosomal constitution. Thus, monopronuclear IVF and ICSI oocytes in which the single pronucleus persists after a second assessment might be used for transfer when they are able to reach the blastocyst stage and when no other embryo is available. Of course, each institution will have to clarify whether this approach should be accompanied by adequate counselling on potential genetic risks and written consent of the patients and whether a preimplantation genetic diagnosis could be offered. Finally, though Mateo et al. [16] discourage from the use of diploid-mosaic blastocysts, it should be considered that a high rate of aneuploidy and mosaicism even occurs in high quality embryos derived from normally fertilized ICSI oocytes [34]. Therefore, the implantation potential of such embryos and possible mechanisms of self-correction of abnormal chromosome complements are topics of future research. Another important question may concern the epigenetic status of fertilized monopronuclear oocytes, particularly whether the interaction between paternal and maternal chromatin is disturbed when they are prematurely enclosed within one pronuclear envelope [12].

## 5. Conclusions

Oocytes in which a single pronucleus persists might be considered for transfer if they reach a good-quality blastocyst stage but this remains an individual decision of the IVF laboratory. More data on the morphologic quality, developmental ability, and genetic constitution of affected embryos are undoubtedly needed before a general consent can be achieved that should include recommendations on counselling of the patients and the role of preimplantation genetic diagnosis. The incidence and significance of early pronuclear fusion events or an immediate enclosure of paternal and maternal chromatin within a single pronuclear envelope might be an interesting topic of future research, particularly in view of genetic and epigenetic implications.

## Figures and Tables

**Table 1:** The genetic constitution of monopronuclear oocytes and resultant developmental stages.

Material	Origin	Number of cases	Method	Cytogenetic constitution			Reference
				Haploid	Diploid	Other	
Karyoplasts	IVF	16	FISH	10 (62.5%)	6 (37.5%)	0	Levron et al. [11]
Zygotes	IVF	20	Cytogenetics/FISH	9 (45.0%)	11 (55.0%)	0	Balakier et al. [13]
Zygotes	IVF	45	Histone methylation	6 (13.3%)	39 (86.7%)	0	van der Heijden et al. [12]
Embryos	IVF	54	Cytogenetics	? (69.0%)	? (13.0%)	? (17.0%)	Plachot et al. [19]
Embryos	IVF	9	Cytogenetics	3 (33.3%)	5 (55.6%)	1 (11.1%)	Jamieson et al. [20]
Embryos	IVF	41	Cytogenetics	5 (12.2%)	33 (80.5%)	3 (7.3%)	Staessen et al. [2]
Embryos	IVF	21	FISH	3 (14.3%)	15 (71.4%)	3 (14.3%)	Sultan et al. [3]
Embryos	IVF	115	FISH	15 (13.0%)	56 (48.7%)	44 (38.3%)	Staessen and van Steirteghem [7]
Embryos	IVF	26	Cytogenetics	6 (23.1%)	19 (73.1%)	1 (3.8%)	Lim et al. [14]
Embryos	IVF	46	FISH	11 (23.9%)	25 (54.3%)	10 (21.7%)	Yan et al. [21]
Blastocysts	IVF	6	FISH	0	6 (100%)	0	Otsu et al. [22]
Zygotes	ICSI	18	Cytogenetics	18 (100%)	0	0	Rosenbusch (unpublished data)
Zygotes	ICSI	28	Cytogenetics	28 (100%)	0	0	Macas et al. [17]
Zygotes	ICSI	33	Histone methylation	23 (69.7%)	10 (30.3%)	0	van der Heijden et al. [12]
Embryos	ICSI	21	FISH	14 (66.7%)	6 (28.6%)	1 (4.8%)	Sultan et al. [3]
Embryos	ICSI	61	FISH	19 (31.2%)	17 (27.9%)	25 (41.0%)	Staessen and van Steirteghem [7]
Embryos	ICSI	24	Cytogenetics	14 (58.3%)	9 (37.5%)	1 (4.2%)	Lim et al. [14]
Embryos	ICSI	73	FISH	23 (31.5%)	23 (31.5%)	27 (37.0%)	Yan et al. [21]
Embryos	ICSI	46	FISH	8 (17.4%)	1 (2.2%)	37 (80.4%)	Mateo et al. [16]
Blastocysts	ICSI	8	FISH	1 (12.5%)	3 (37.5%)	4 (50.0%)	Mateo et al. [16]
Embryos	IVF/ICSI	95	FISH	29 (30.5%)	37 (38.9%)	29 (30.5%)	Liao et al. [15]
Blastocysts	IVF/ICSI	59	FISH	0	46 (78.0%)	13 (22.0%)	Liao et al. [15]

Embryos comprise developing or arrested cleavage stages including the morula. The category “Haploid” may contain deviations from the exact chromosome count of 23 and haploid-mosaic cells. Also, the category “Diploid” may contain deviations from the exact chromosome count of 46 and diploid-mosaic cells (see Results). “Other” cytogenetic constitutions include polyploid, mosaic, complex, and chaotic cases. ?: absolute numbers not indicated.

**Table 2:** A brief summary of our cytogenetic analysis of monopronuclear oocytes obtained after ICSI^a^.

Number of patients	16	
Number of oocytes fixed	20	
Number of analyzable oocytes	18	
Number of diploid oocytes	0	
Number of haploid oocytes	18 (100%)	
Haploid abnormal:		
Hypohaploidy	2 (11.1%)	22,X,−B
		22,X,−D
Hyperhaploidy	2 (11.1%)	24,X,+C
		24,X,+E

^a^Previously unpublished data.

**Table 3:** The origin of diploidy in monopronuclear oocytes and ensuing developmental stages.

Material	Origin	Method	Diploid cells	Heteroparental cells	Reference
Karyoplasts	IVF	Y-detection by FISH	6	4 (66.7%)	Levron et al. [11]
Zygotes	IVF	Histone methylation patterns^a^	39	39 (100%)	van der Heijden et al. [12]
Embryos	IVF	Cytogenetics/karyotyping	5	2 (40.0%)	Jamieson et al. [20]
Embryos	IVF	Y-detection by FISH	15	9 (60.0%)	Sultan et al. [3]
Embryos	IVF	Y-detection by FISH	56	25 (44.6%)	Staessen and van Steirteghem [7]
Embryos	IVF	Y-detection by FISH	25	15 (60.0%)	Yan et al. [21]
Blastocysts	IVF	Y-detection by FISH	6	4 (66.7%)	Otsu et al. [22]
Zygotes	ICSI	Histone methylation patterns^a^	10	10 (100%)	van der Heijden et al. [12]
Embryos	ICSI	Y-detection by FISH	6	1 (16.7%)	Sultan et al. [3]
Embryos	ICSI	Y-detection by FISH	17	6 (35.3%)	Staessen and van Steirteghem [7]
Embryos	ICSI	Y-detection by FISH	23	12 (52.2%)	Yan et al. [21]

^a^Note that this is a nongenetic method that distinguishes maternal and paternal chromatin independent of the occurrence of specific chromosomes. Under the assumption that X- and Y-spermatozoa participate equally in fertilization, the figures obtained by detection of a Y-chromosome should be doubled [9] and then yield percentages of heteroparental cells that are comparable to the findings of van der Heijden et al. [12].
